# Ebselen abolishes vascular dysfunction in influenza A virus-induced exacerbations of cigarette smoke-induced lung inflammation in mice

**DOI:** 10.1042/CS20211090

**Published:** 2022-04-21

**Authors:** Kurt Brassington, Stanley M.H. Chan, Simone N. De Luca, Aleksandar Dobric, Suleman A. Almerdasi, Kevin Mou, Huei Jiunn Seow, Osezua Oseghale, Steven Bozinovski, Stavros Selemidis, Ross Vlahos

**Affiliations:** School of Health and Biomedical Sciences, RMIT University, Bundoora, VIC 3083 Australia

**Keywords:** acute exacerbations of chronic obstructive pulmonary disease, antioxidant, cardiovascular disease, cigarette smoke, endothelium, vascular dysfunction

## Abstract

People with chronic obstructive pulmonary disease (COPD) are susceptible to respiratory infections which exacerbate pulmonary and/or cardiovascular complications, increasing their likelihood of death. The mechanisms driving these complications remain unknown but increased oxidative stress has been implicated. Here we investigated whether influenza A virus (IAV) infection, following chronic cigarette smoke (CS) exposure, worsens vascular function and if so, whether the antioxidant ebselen alleviates this vascular dysfunction. Male BALB/c mice were exposed to either room air or CS for 8 weeks followed by inoculation with IAV (Mem71, 1 × 10^4.5^ pfu). Mice were treated with ebselen (10 mg/kg) or vehicle (5% w/v CM-cellulose in water) daily. Mice were culled 3- and 10-days post-infection, and their lungs lavaged to assess inflammation. The thoracic aorta was excised to investigate endothelial and smooth muscle dilator responses, expression of key vasodilatory and oxidative stress modulators, infiltrating immune cells and vascular remodelling. CS increased lung inflammation and caused significant vascular endothelial dysfunction, which was worsened by IAV infection. CS-driven increases in vascular oxidative stress, aortic wall remodelling and suppression of endothelial nitric oxide synthase (eNOS) were not affected by IAV infection. CS and IAV infection significantly enhanced T cell recruitment into the aortic wall. Ebselen abolished the exaggerated lung inflammation, vascular dysfunction and increased T cell infiltration in CS and IAV-infected mice. Our findings showed that ebselen treatment abolished vascular dysfunction in IAV-induced exacerbations of CS-induced lung inflammation indicating it may have potential for the treatment of cardiovascular comorbidities seen in acute exacerbations of COPD (AECOPD).

## Introduction

Chronic obstructive pulmonary disease (COPD) is an incurable disease characterised by a progressive airflow limitation and irreversible decline in lung function. COPD is currently the third leading cause of death globally and claims the lives of over 3 million people annually [1,2]. The debilitating respiratory symptoms associated with COPD are caused by abnormalities to the airways driving alveolar destruction, primarily due to exposure to noxious gases and particulate matter inhaled from cigarette smoke (CS) [3]. COPD patients often experience various extrapulmonary comorbid diseases, with cardiovascular disease (CVD) causing approximately 50% of all COPD-related deaths [3,4]. People with COPD experience up to four episodes of worsened symptoms termed acute exacerbations of COPD (AECOPD) annually [3,5], with a study by Echevarria et al. showing that of 2417 patients released from hospital, 936 either died or were readmitted within 90 days of discharge [6]. AECOPD are predominantly due to viral and/or bacterial infections, which worsen the severity of both the pulmonary and cardiovascular manifestations associated with this disease, profoundly impacting the quality of life of these patients and drastically increasing medical treatment costs and hospital admissions [7–9]. Viral exacerbations account for up to 60% of all AECOPD, with both rhinovirus and influenza A virus (IAV) being the most frequently observed invading pathogens [7].

It is understood that COPD and vascular diseases share an array of common risk factors and mechanisms, these include; ageing, smoking status, inflammation, and oxidative stress [10–13]. We and others have shown that CS-induced oxidative stress is a major driver of respiratory pathologies commonly seen in COPD [14–16] and CVD comorbidities [13]. We have also shown that targeting CS-induced oxidative stress can reduce lung inflammation and vascular dysfunction in mice [13,16]. There is mounting evidence suggesting that the increased mortality rate observed in AECOPD may be attributed to acute vascular diseases, resulting from heightened endothelial inflammation, vascular remodelling, and atherosclerosis [17–19]. During AECOPD there is also a heightened oxidative burden resulting from increased reactive oxygen species (ROS) production and depletion of innate antioxidant mechanisms [20,21], which may perturb the normal function of endothelial cells; which line the interior of blood vessels and are vulnerable to oxidative stress [22]. Antus et al*.* investigated the activity of major antioxidant enzymes in AECOPD patients that required hospitalisation and found that increased sputum levels of antioxidant enzymes such as glutathione peroxidase (GPx) confer a degree of protection against the pulmonary oxidative damage during exacerbations [20]. However, little is known about the effects of exogenous antioxidant supplementation in the context of comorbid disease incidence and severity in AECOPD.

In the present study, we investigated whether IAV infection exacerbates CS-induced vascular dysfunction in mice and whether targeting oxidative stress with the GPx mimetic ebselen preserves vascular function in the context of AECOPD.

## Methods

### Animals

All animal experimentation was performed at the RMIT University Animal Facility and conducted in compliance with the Australian Code of Practice for the Care of Experimental Animals and RMIT University Animal Ethics approval (Application Number 1533). Male BALB/c mice were sourced from the Animal Resource Centre Pty. Ltd (Perth, Australia) at 7 weeks of age and housed at 21°C in micro-isolator cages (Able Scientific, Australia) with a 12-h light/dark cycle. Mice were acclimatised for a week prior to being used for experiments. All mice had unrestricted access to standard mouse chow (Glen Forest Speciality Foods, Australia) and water.

### CS exposure and drug administration

Mice were placed into an 18L Perspex Chamber (The Plastic Man, Australia) and exposed to CS, produced by 9 Winfield Red Cigarettes (16 mg tar, 1.2 mg nicotine, 16 and 15 mg CO, total particulate matter 419 mg m^−3^, Philip Morris, Moorabbin, Australia) per day/5 days a week (Monday–Friday) for 8 weeks with a 6 ml/second timed draw (60 ml total draw volume) as previously described [13]. These mice (*n*=14 per group) were subject to three CS sessions a day, 2 h apart, with three cigarettes delivered per session. Sham-exposed animals were placed in an identical Perspex box, however they did not receive CS. For the ebselen studies, treatments were administered daily via oral gavage 1 h prior to the initial CS exposure. Ebselen (Cayman Chemical, USA) was administered at a pre-established dose of 10 mg.kg^−1^ in 5% w/v CarboxyMethyl-cellulose (CM-cellulose) dissolved in water (Sigma–Aldrich, U.S.A.), with vehicle treated mice subjected to 5% CM-cellulose alone as previously described [16,23–26]. Upon completion of the 8-week CS protocol, mice were intranasally inoculated with Mem71 IAV (provided by Dr Andrew Jarnicki and Prof Gary Anderson, University of Melbourne, Australia) (1 × 10^4.5^ pfu) or diluent (Dil; sterile PBS) and culled 3- and 10-days post-infection. The body weight of all mice was monitored every alternate day. This murine model of CS exposure recapitulates the key traits of human AECOPD, including pulmonary inflammation and oxidative stress as well as the ability to induce various comorbid diseases including CVD and skeletal muscle wasting [11,13,27].

### Bronchoalveolar lavage and lung tissue collection

Animals were killed via intraperitoneal injection with sodium pentobarbitone (240 mg.kg^−1^; Virbac, Australia). An *in-situ* lavage was then conducted on the lungs via surgical tracheotomy and flushed with 0.4 ml of chilled sterile PBS, followed by 0.3 ml PBS washes thrice with approximately 1 ml of bronchoalveolar lavage (BAL) fluid (BALF) being retrieved per mouse as published previously [11,13,16,28]. Total viable cell counting was conducted on the BALF using fluorescent acridine orange (Invitrogen, U.S.A.) and a standard Neubauer haemocytometer using an Olympus BX53 microscope (Olympus, Japan) as previously described [11,13,16,28].

#### Total and differential cell counting

Quantification of immune cell populations within the BALF was conducted using cytospots containing ∼5 × 10^4^ cells/slide (cytocentrifuged cell spots, Shandon Cytospin 3, 18.06 g, 10 min), dried then subjected to fixation using Shandon™ Kwik-Diff^®^ Fixative Reagent 1 (Thermo Fischer Scientific, U.S.A.), then Hemacolour® Solutions 2 and 3 (Merck, U.S.A.). Thiazine and Eosin differential stain was completed as per the manufacturer’s instructions. Cells were differentiated using standard morphological criteria to distinguish macrophages, neutrophils, and lymphocytes with >500 cells counted per sample.

### Vessel myography

To investigate the effect of CS exposure, virus infection and ebselen treatment on vascular function, the thoracic aorta was excised from the chest cavity, placed in a Petri dish containing carbogen-bubbled (95% O_2_, 5% CO_2_) Krebs buffer (composition in mmol/l: NaCl 119, KCl 4.7, MgSO_4_ 1.17, NaHCO_3_ 25, KH_2_PO_4_ 1.18, CaCl_2_ 2.5, glucose 5.5) and dissected free of perivascular fat and connective tissue. Aortae were then cut into 2-mm rings and subjected to vessel myography (*ex vivo* functional testing) using a 4-channel myograph unit (Danish Myo Technology (DMT) A/S, Model 610M), as previously published [13].

#### Immunohistological staining for vascular endothelial nitric oxide synthase, CD3+ T cells, peroxynitrite expression, and aortic remodelling

Vascular endothelial nitric oxide synthase (eNOS/NOS3) expression, a key enzyme responsible for the production of nitric oxide (NO) within the endothelium, was measured using a NOS3 antibody (Thermo Fisher Scientific, U.S.A., 1:100 dilution). Vascular peroxynitrite was quantified using antibody staining against 3-Nitrotyrosine (3-NT) (Thermo Fisher Scientific, 1:100 dilution), which is the product of NO and superoxide. T cell number was determined using Cluster of Differentiation 3 (CD3)-specific monoclonal antibody (Abcam, U.K., 1:100 dilution). Aortic wall remodelling was assessed using Masson’s Trichrome Stain (Sigma–Aldrich, procedure number HT15) as per the manufacturer’s instructions.

All fixation of tissue, histology and immunofluorescent staining on the thoracic aorta was completed as published [13], then imaged using an Olympus slide scanner VS120-SS (Olympus, Japan). The expression of both 3-NT and eNOS was completed using Olympus cellSens Dimension™ desktop software, calculating Object Area Fraction ROI (%) (version 1:18, Olympus Corporation) in the vascular endothelial layer. CD3+ T cell counting was conducted manually with all analysis being completed in a blinded manner. Aortic wall remodelling was also quantified using cellSens Dimension™ desktop software (Olympus, Japan) which measures both aortic wall thickness and collagen deposition as fraction ROI %.

#### Data and statistical analysis

All data are presented as mean ± standard error of the mean (SEM) with statistical differences determined by analysis of variance (ANOVA) [29] and where appropriate, Tukey’s post-hoc multiple comparison tests were conducted between treatment groups. Where data were influenced by two factors, data were analysed using a two-way ANOVA, with all statistical analyses completed using GraphPad Prism™ (version 9 GraphPad software® for Microsoft Windows®, U.S.A.), with *P*<0.05 deemed significant in all cases. All statistical and data analyses were completed with groups of equal size, blinded analysis and randomisation were taken into consideration during the experimental design. Statistical analysis was only undertaken where each group was equal to or greater than *n*=5; where *n* was the number of independent values and not technical replicates.

## Materials

Some key compounds/consumables including the Winfield Red Cigarettes were obtained from Phillip Morris, Australia; ebselen from Cayman Chemical, U.S.A., and carboxymethyl cellulose from Sigma–Aldrich, U.S.A. All materials used throughout the present study were previously published in Brassington et al. (2021), with the exception of the following: anti CD-3 monoclonal antibody RRID: AB16669 (Abcam, U.K.); Goat anti-rabbit IgG (H&L) secondary antibody, Alexa Flour 488 RRID: AB150077 (Abcam, U.K.); Fluoromount-G^TM^ DAPI (Thermo Fisher Scientific, U.S.A.).

## Results

### Lung inflammation is worsened following IAV infection in CS-exposed mice

**Figure 1:**
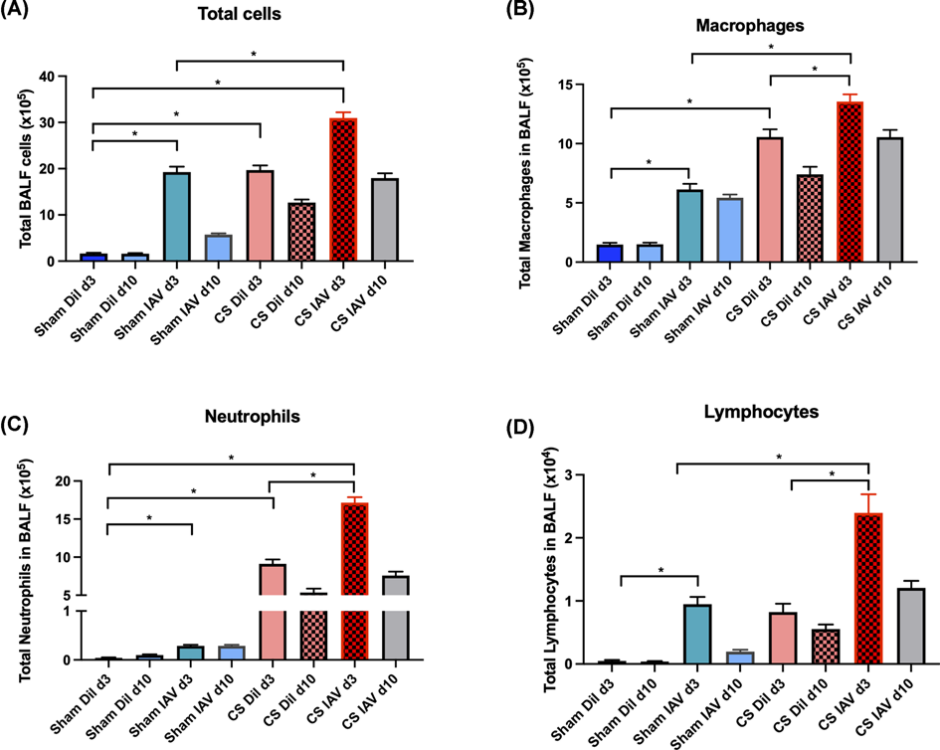
Exposure to CS increases immune cell recruitment to the lungs of Balb/c mice, which is worsened by IAV infection The lungs of CS or room air ± IAV-exposed mice were lavaged for the quantification of total cell (**A**), macrophages (**B**), neutrophils (**C**) and lymphocytes (**D**) populations. Data expressed as mean ± SEM (*n*=14 mice per group). BALF cellular composition was determined by differential counting of cytospots. Data analysed by two-way ANOVA and Tukey’s multiple comparisons with significance being denoted by * indicating *P<*0.05 between treatment groups, respectively.

Chronic CS exposure caused significant immune cell recruitment into the BALF at 3 and 10 days post-last CS exposure in the diluent-treated animals, which was attributed to an increase in both macrophages and neutrophils (Figure 1). IAV infection alone also caused a significant increase in BALF total cell infiltration in sham-exposed mice at d3, however, this was not observed at d10 post-infection indicating viral clearance. This increase in immune cells was attributed to enhanced macrophage, neutrophil, and lymphocyte recruitment (Figure 1). Interestingly, CS in conjunction with IAV infection caused a further enhancement in lung immune cell infiltration, when compared with the uninfected (i.e. diluent) controls. This exacerbated phenotype was observed across macrophage, neutrophil, and lymphocyte populations in the BALF (Figure 1). However, at the d10 post-infection time point, the number of total cells returned to baseline for each group when compared with their respective diluent control.

### Aortic endothelial dysfunction is worsened following IAV infection in CS-exposed mice

**Figure 2:**
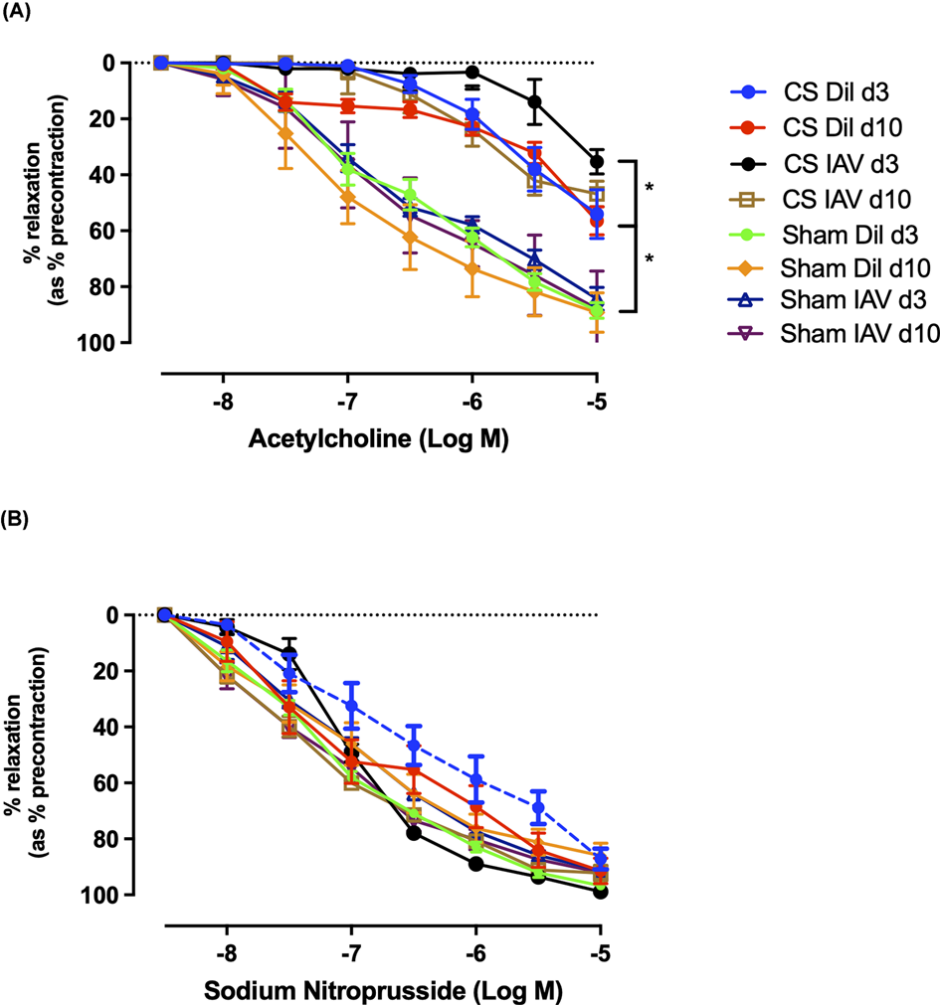
Viral infection exacerbates endothelial dysfunction following exposure to CS Cumulative concentration–response curves to ACh (**A**) and SNP (**B**) (1 × 10^−8^ to 1 × 10^−5^ M) to analyse endothelium-dependent and smooth muscle-dependent vasodilation in mouse thoracic aorta, respectively (*n*=8) following either chronic CS or sham exposure in either IAV-infected or diluent (Dil)-treated mice. Results expressed as percentage of mean relaxation relative to initial preconstriction ± SEM. * indicates statistical significance *P*<0.05 between datasets by two-way ANOVA with Tukey’s multiple comparisons.

We have previously shown that chronic CS exposure impairs vascular function in mice [13]. Therefore, the effect of IAV infection following chronic CS exposure in the vasculature was explored to determine if there is a further worsening of endothelial function. Aortae taken from CS-exposed diluent-treated mice showed significant endothelial dysfunction in response to acetylcholine (ACh; 10^−8^ to 10^−5^ M) at days 3 and 10 (maximal relaxation (R_MAX_) ∼50%, Figure 2A) post-infection, when compared with the sham-treated groups (R_MAX_ ∼80%, *n*=8, *P*<0.05). Sham-exposed IAV-infected mice did not have impaired vascular function in response to ACh at day 3 or day 10 (R_MAX_ ∼80%, *n*=8). However, IAV-infected mice exposed to CS showed further impaired endothelial dysfunction at the day 3-time point (peak infection) (R_MAX_ 35%, *n*=8, *P*<0.05), however, at day 10 (resolution phase) there were no differences observed between CS + IAV and CS + diluent-treated groups. There were no alterations in vascular smooth muscle function with vessels showing an ∼80–90% R_MAX_ to sodium nitroprusside (SNP) irrespective of either CS or IAV status (Figure 2B). These findings indicate that CS exposure causes endothelial dysfunction that is exacerbated following IAV infection, whilst having no effect on smooth muscle dilation.

### CS exposure alters vascular eNOS expression, enhances oxidative stress, and promotes immune cell recruitment upon IAV infection

**Figure 3:**
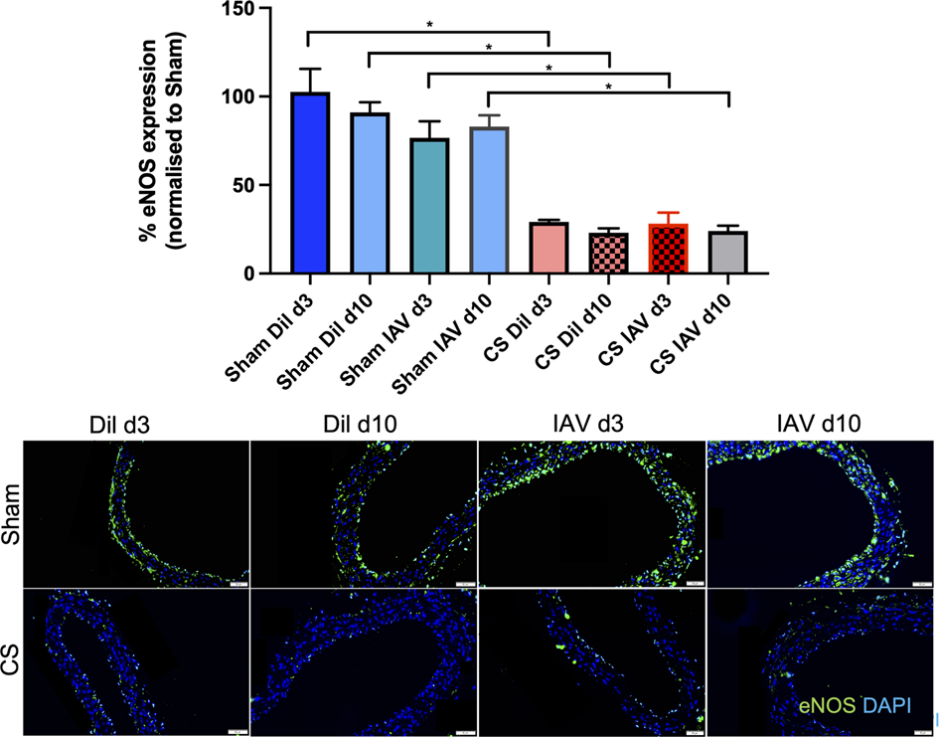
CS exposure causes the depletion of eNOS in the thoracic aorta irrespective of IAV infection Green staining indicates eNOS-specific expression and blue staining is the nucleus-specific stain DAPI (4′,6-diamidino-2-phenylindole, dilactate). eNOS expression is normalised to the negative control and expressed as percentage change relative to the sham vehicle-treated group. Scale bar represents 50 μM. Data expressed as mean ± SEM (*n*=5 mice per group) and analysed by two-way ANOVA with Tukey’s multiple comparisons; * indicates statistical significance *P*<0.05.

**Figure 4:**
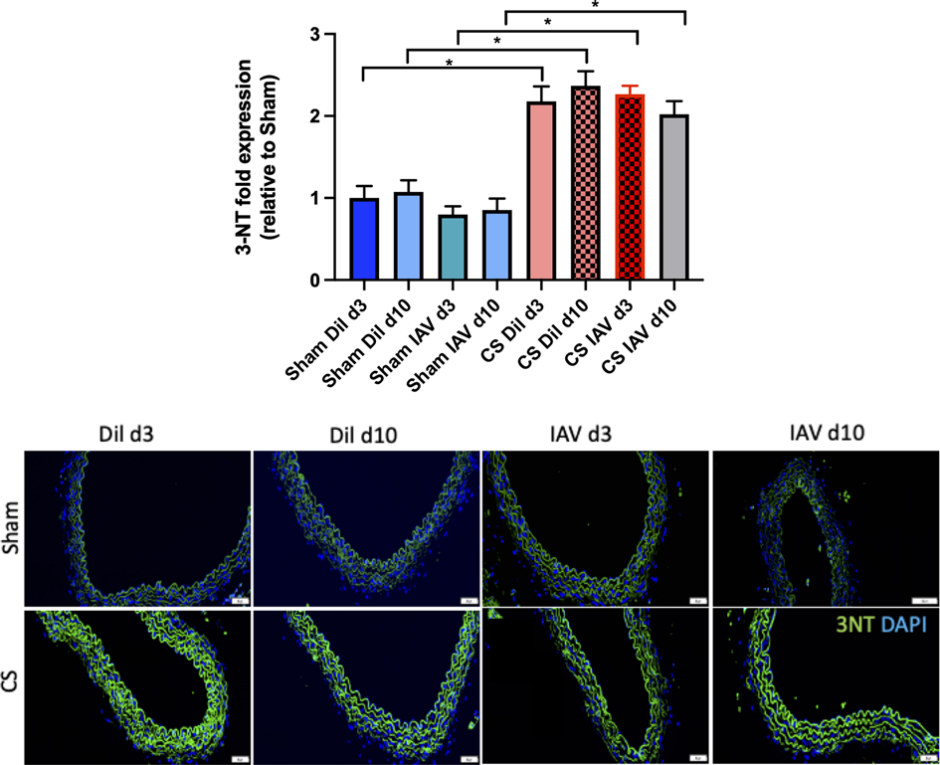
Exposure to CS promotes significant oxidative stress marked by the accumulation of 3-NT in the thoracic aorta in both stable and IAV-infected mice Green staining indicates 3-NT-specific expression and blue staining is the nucleus-specific stain DAPI. 3-NT expression is normalised to the negative control and expressed as fold change relative to the sham vehicle-treated group. Scale bar represents 50 μM. Data expressed as mean ± SEM (*n*=5 mice per group) and analysed by two-way ANOVA with Tukey’s multiple comparisons; * indicates statistical significance *P*<0.05.

Mice exposed to CS showed a significant reduction in the expression of eNOS (∼60%) in the aorta at d3 and d10 (*n*=6, *P<*0.05), when compared with sham diluent-treated control mice irrespective of IAV infection, whereas there was no reduction in eNOS expression in sham-exposed mice infected with IAV (Figure 3). Vascular oxidative stress was worsened in response to CS as evidenced by the ∼1.5 fold up-regulation of 3-NT on both d3 and d10 when compared with sham-treated controls (Figure 4, *n*=5, *P*<0.05), however, IAV infection had no added effect on 3-NT expression. In addition, IAV infection alone had no effect on 3-NT expression (Figure 4).

**Figure 5:**
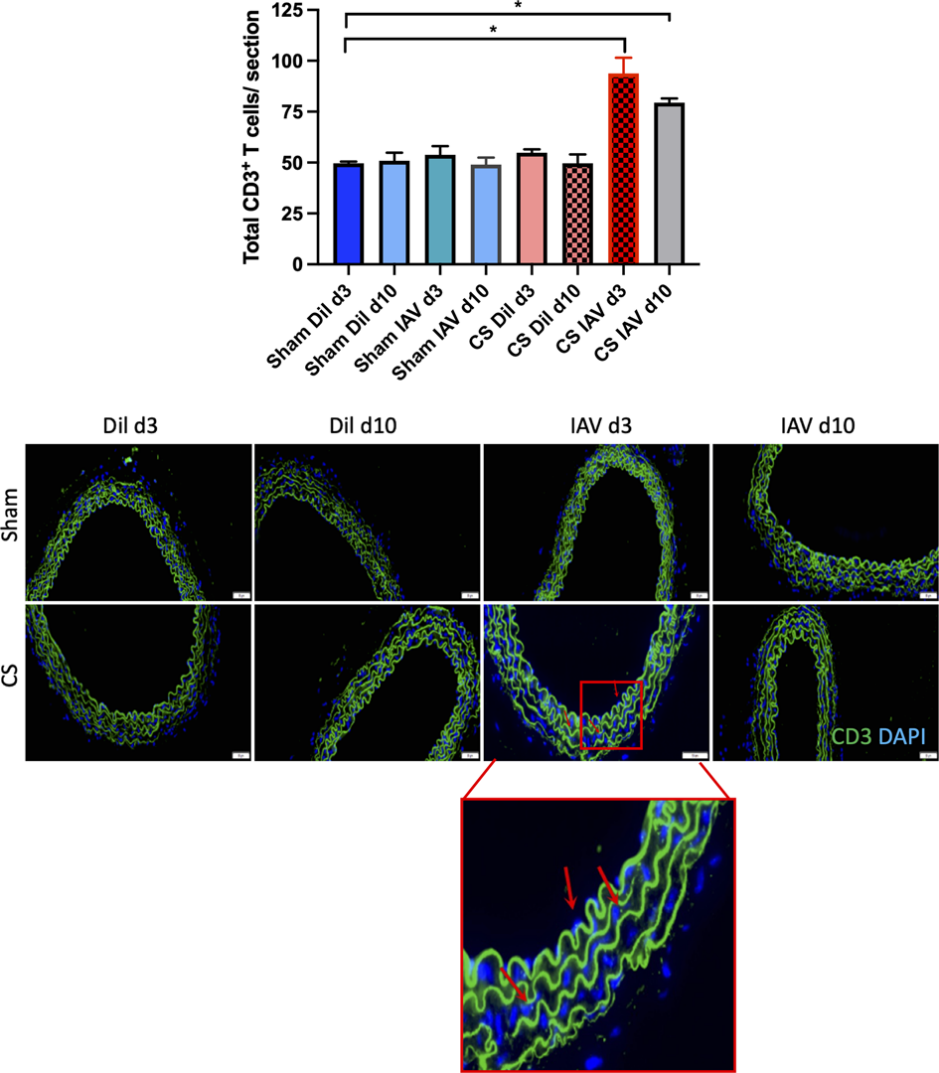
CD3 T cell recruitment is increased only in the thoracic aorta of IAV-infected mice with chronic CS exposure Green staining indicates CD3 expressing T cells and blue staining denotes the nuclear counterstain, DAPI. Representative photographs of immunofluorescent staining across all treatments. CD3+ T cells were manually counted, and raw values plotted. Scale bar represents 50 μM. Data expressed as mean ± SEM (*n*=5 mice per group) and analysed by two-way ANOVA with Tukey’s multiple comparisons; * indicates statistical significance *P*<0.05.

T cell recruitment into the aortic wall was then investigated, as these cells play a crucial role in orchestrating the adaptive immune response to foreign pathogens such as the IAV. CS exposure or IAV infection alone had no effect on the recruitment of CD3+ T cells on both d3 and d10 (Figure 5). However, IAV infection following CS exposure caused a significant increase in the number of infiltrating CD3+ T cells into the aortic wall on both d3 and d10 when compared with the respective sham-exposed control group (Figure 5, *n*=5, *P*<0.05). Interestingly, there was no increase in the number of infiltrating T cells in the aortae of sham + IAV-infected mice, indicating that this is purely a CS-dependent phenomenon.

### Ebselen treatment alters BALF cell populations in CS-exposed mice infected with IAV

**Figure 6:**
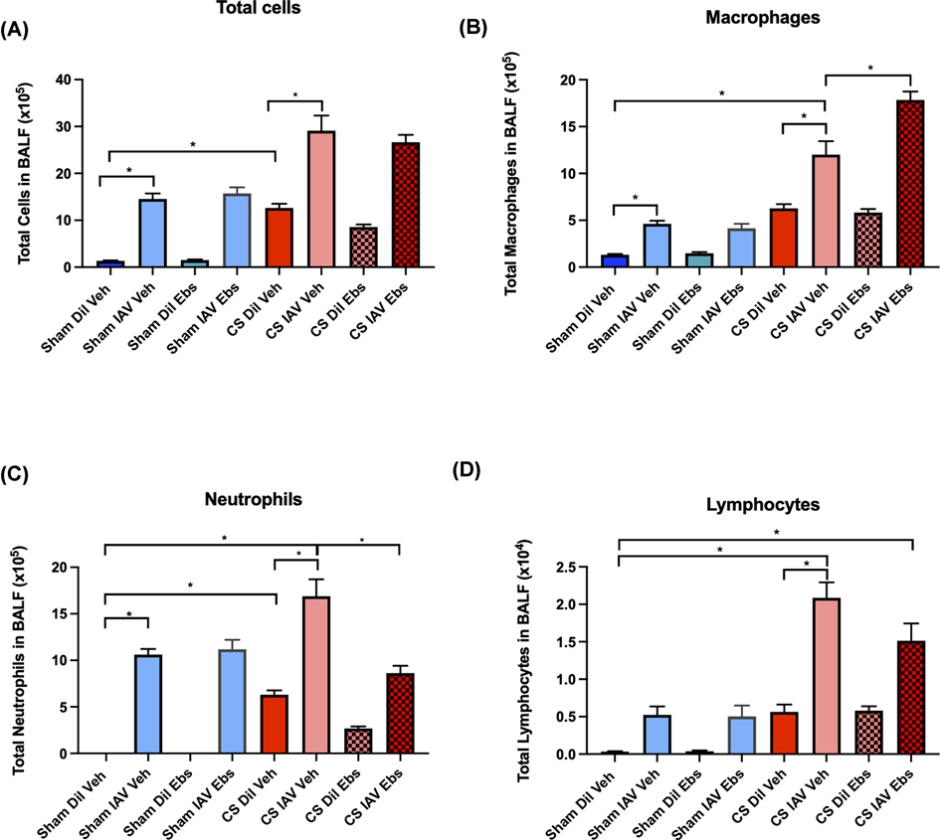
Ebselen alters immune cell populations in the BAL Fluid of mice exposed to CS ± IAV infection The lungs of CS or room air ± IAV-exposed mice were lavaged for the quantification of total cell (**A**), macrophages (**B**), neutrophils (**C**) and lymphocytes (**D**) populations. Data expressed as mean ± SEM (*n*=14 mice per group). BALF cellular composition was determined by differential counting of cytospots. Data analysed by two-way ANOVA and Tukey’s multiple comparisons with significance being denoted by * indicating *P<*0.05 between treatment groups, respectively.

Consistent with Figure 1, CS + Dil treatment caused a significant increase in BALF total cells when compared with the sham + Dil-treated controls (Figure 6, *P*<0.05). IAV infection alone also caused a significant increase in total cell infiltration into the BALF when compared with their uninfected counterparts (*P*<0.05). However, CS + IAV-infected mice showed a significant exacerbated phenotype, when compared with their sham-exposed counterparts or either CS or IAV alone. The further increase in total cells within the BALF of these CS + IAV animals was attributed to significantly amplified macrophage, neutrophil, and lymphocyte recruitment into the lungs. Treatment with ebselen had no effect on BALF total cells and lymphocytes in the CS + IAV group, however the number of infiltrating neutrophils was significantly reduced (Figure 6C). Conversely, CS + IAV + Ebselen-treated mice showed a significant increase in the number of macrophages within the BALF when compared with the CS + IAV + Veh-treated group (Figure 6B, *P*<0.05).

### Ebselen prevents endothelial dysfunction caused by CS and IAV infection

**Figure 7:**
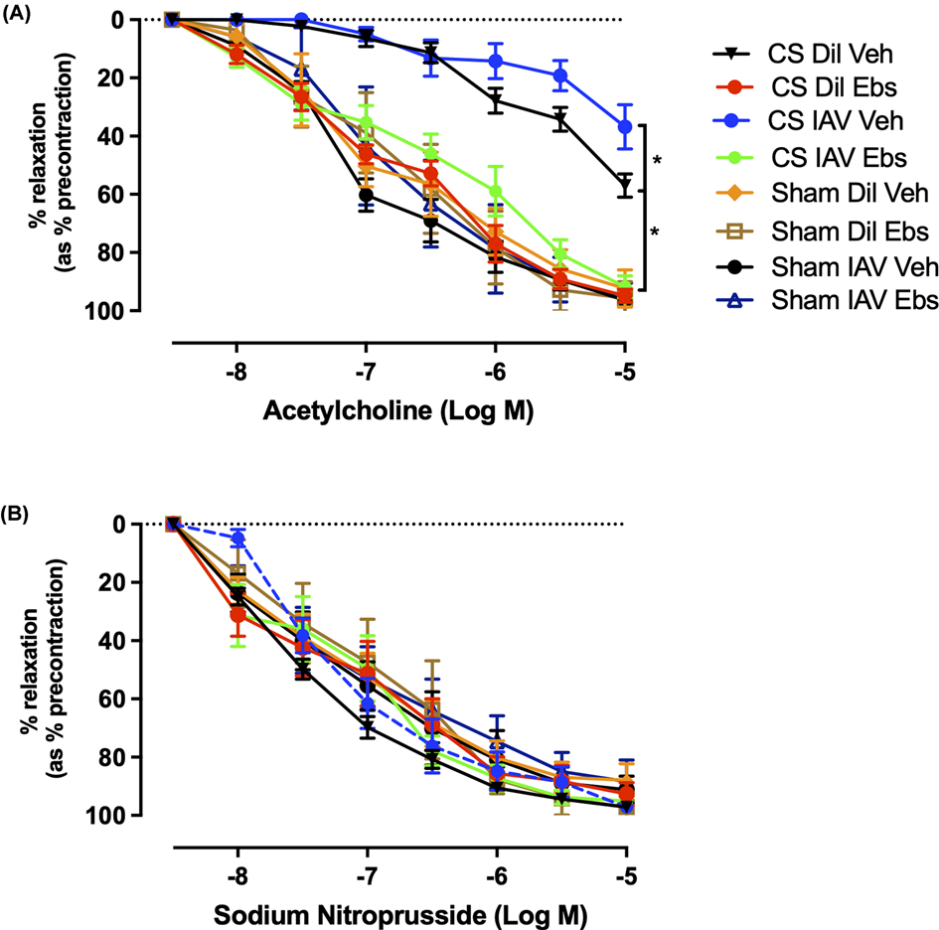
Ebselen treatment preserves vascular endothelial function in CS-exposed mice irrespective of IAV infection Cumulative concentration–response curves to ACh (**A**) and SNP (**B**) (1 × 10^−8^ to 1 × 10^−5^ M) to analyse endothelium-dependent and smooth muscle-dependent vasodilation in mouse thoracic aorta, respectively (*n*=8) following either chronic CS or sham exposure in either IAV-infected or diluent (Dil)-treated mice subject to either ebselen (Ebs) or vehicle (Veh) treatment. Results expressed as percentage mean relaxation relative to initial preconstriction ± SEM. * indicates statistical significance *P*<0.05 between datasets by two-way ANOVA with Tukey’s multiple comparisons.

Based on our findings, endothelial dysfunction is worsened during IAV infection, with studies showing that this leads to an increased likelihood of cardiovascular events such as myocardial infarction (MI) and stroke [30,31]. We have previously established that modulation of oxidative stress in the vasculature can preserve vascular function against the deleterious effects of CS-driven COPD [13], however, whether this preservative effect is upheld during AECOPD is yet to be investigated. Thus, we next examined the effects of ebselen, a GPx-1 mimetic, on endothelial dysfunction caused by CS and IAV infection. Sham-exposed mice showed an ∼90% R_MAX_ in response to ACh with both viral infection and/or ebselen administration having no effect on endothelial function (Figure 7A). Mice exposed to CS + Dil + Veh showed a significant impairment (50% R_MAX,_ *P*<0.05), when compared with their sham-exposed counterparts. In the CS + IAV + Veh mice, endothelial dysfunction was further worsened in response to ACh (35% R_MAX,_ *P*<0.05). Ebselen administration completely prevented the endothelial dysfunction observed in CS-exposed mice, irrespective of IAV infection (∼90% R_MAX_). Meanwhile, endothelium-independent vasodilation to SNP was unaltered in CS or IAV infected mice irrespective of ebselen treatment (Figure 7B), with ∼90% R_MAX_ observed in all treatment groups.

### Ebselen reduces vascular oxidative stress, inflammation, and structural remodelling whilst preserving eNOS bioavailability in CS-exposed mice infected with IAV

**Figure 8:**
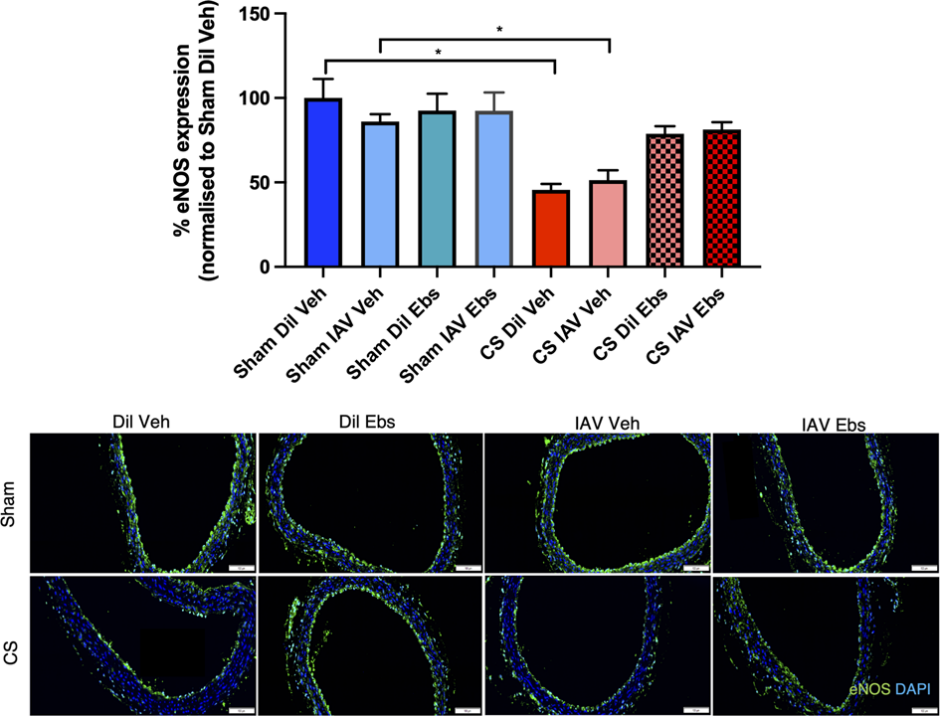
Ebselen treatment prevents the down-regulation of eNOS in the thoracic aorta of CS-exposed mice irrespective of IAV infection Green staining indicates eNOS-specific expression and blue staining is the nucleus-specific stain DAPI (4′,6-diamidino-2-phenylindole, dilactate). eNOS expression is normalised to the negative control and expressed as fold percentage change relative to the sham diluent vehicle-treated group. Scale bar represents 50 μM. Data expressed as mean ± SEM (*n*=5 mice per group) and analysed by two-way ANOVA with Tukey’s multiple comparisons; * indicates statistical significance *P*<0.05.

**Figure 9:**
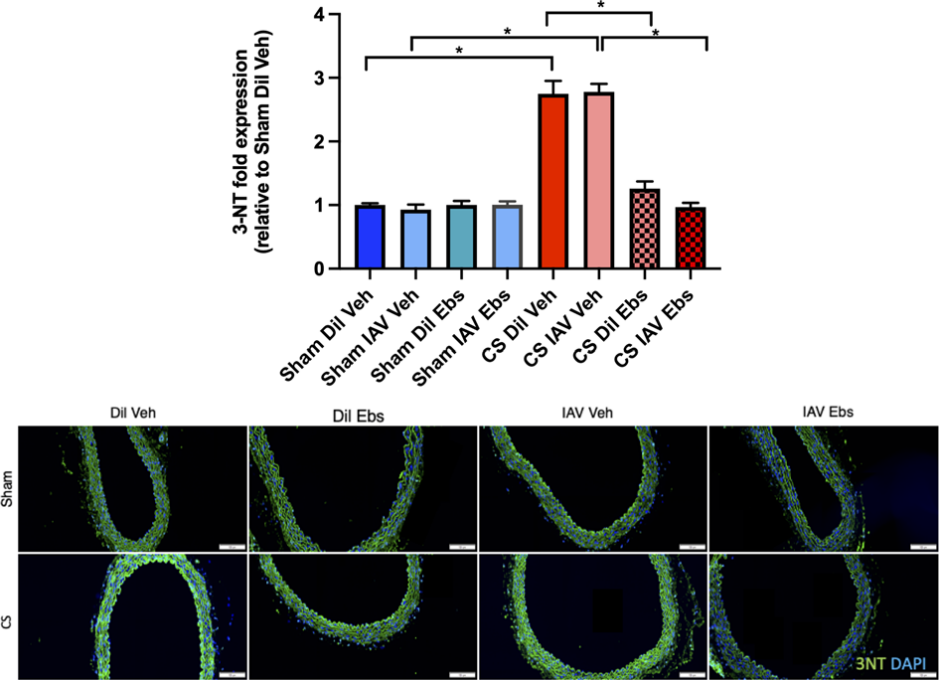
Treatment with ebselen prevents the CS-induced increase in 3-NT expression within the thoracic aorta, which is unaltered during IAV infection Green staining indicates 3-NT-specific expression and blue staining is the nucleus-specific stain DAPI. 3-NT expression is normalised to the negative control and expressed as fold change relative to the sham diluen t vehicle-treated group. Scale bar represents 50 μM. Data expressed as mean ± SEM (*n*=5 mice per group) and analysed by two-way ANOVA with Tukey’s multiple comparisons; * indicates statistical significance *P*<0.05.

Comparable with the data in Figure 3, vehicle-treated CS-exposed mice showed significantly reduced eNOS bioavailability by ∼50%, irrespective of IAV infection (Figure 8). In sham-exposed mice, neither IAV nor ebselen treatment affected aortic eNOS levels (Figure 8). Ebselen-treated mice exposed to CS had completely preserved levels of eNOS irrespective of IAV infection, indicating that ebselen treatment prevented the ablation of eNOS expression following both CS exposure and IAV infection. Aortic expression of 3-NT, was then quantified with vessels taken from sham-exposed mice showing no differences in 3-NT levels in response to either IAV infection and/or ebselen treatment (Figure 9). However, CS + Veh mice had significantly increased aortic 3-NT levels, although treatment with ebselen suppressed this enhanced oxidative stress irrespective of viral infection.

**Figure 10:**
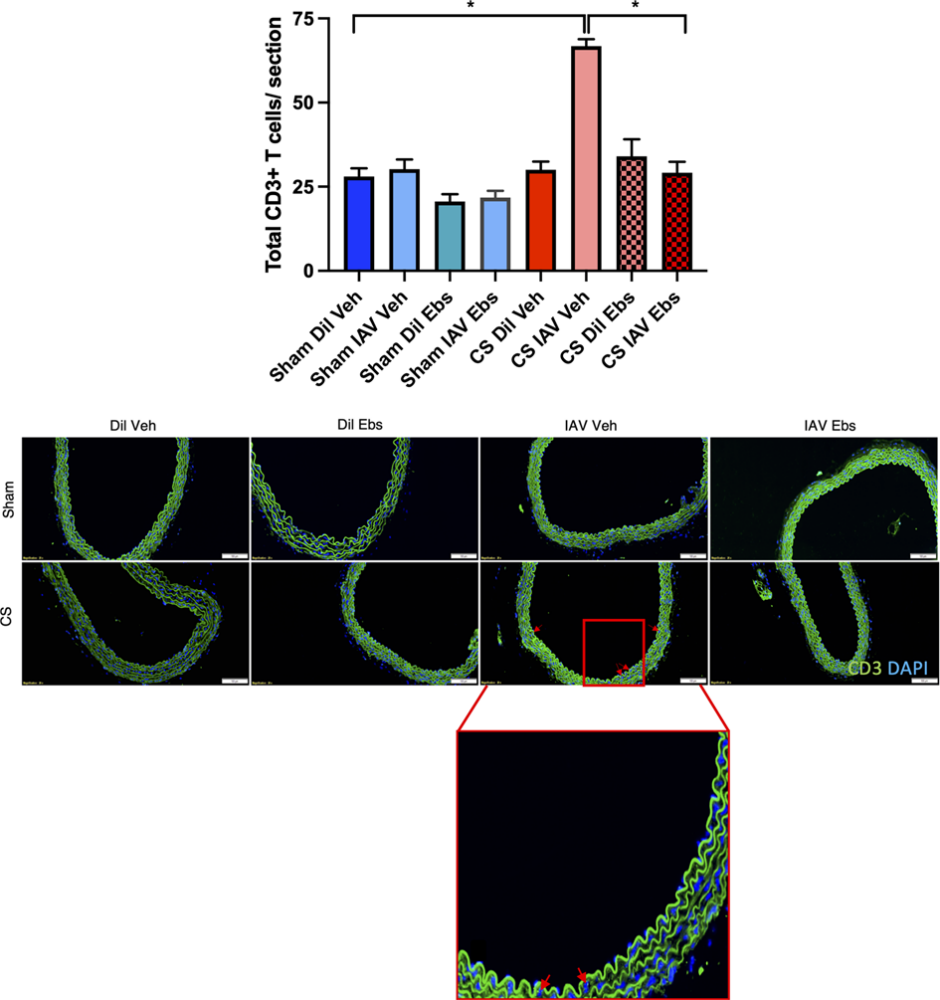
Antioxidant treatment with ebselen prevents vascular CD3+ T cell recruitment into the aortic wall of IAV-infected CS-exposed mice Green staining indicates CD3-expressing T cells and blue staining denotes the nuclear counterstain, DAPI. Representative photographs of immunofluorescent staining across all treatments. CD3+ T cells were manually counted, and raw values plotted. Scale bar represents 50 μM. Data expressed as mean ± SEM (*n*=5 mice per group) and analysed by two-way ANOVA with Tukey’s multiple comparisons; * indicates statistical significance *P*<0.05.

Given that T cell recruitment was significantly enhanced upon viral infection in CS-exposed mice (Figure 6), we then investigated whether ebselen would alter T cell migration into the aortic wall. CS + IAV + Veh-treated mice showed a significant increase in the number of infiltrating CD3+ T cells into the aortic wall. Furthermore, ebselen treatment markedly attenuated the number of CD3+ T cells in the aortic wall of CS + IAV-infected mice (Figure 10, *P*<0.05), indicating that the nature of this recruitment is likely to be oxidant-mediated.

**Figure 11:**
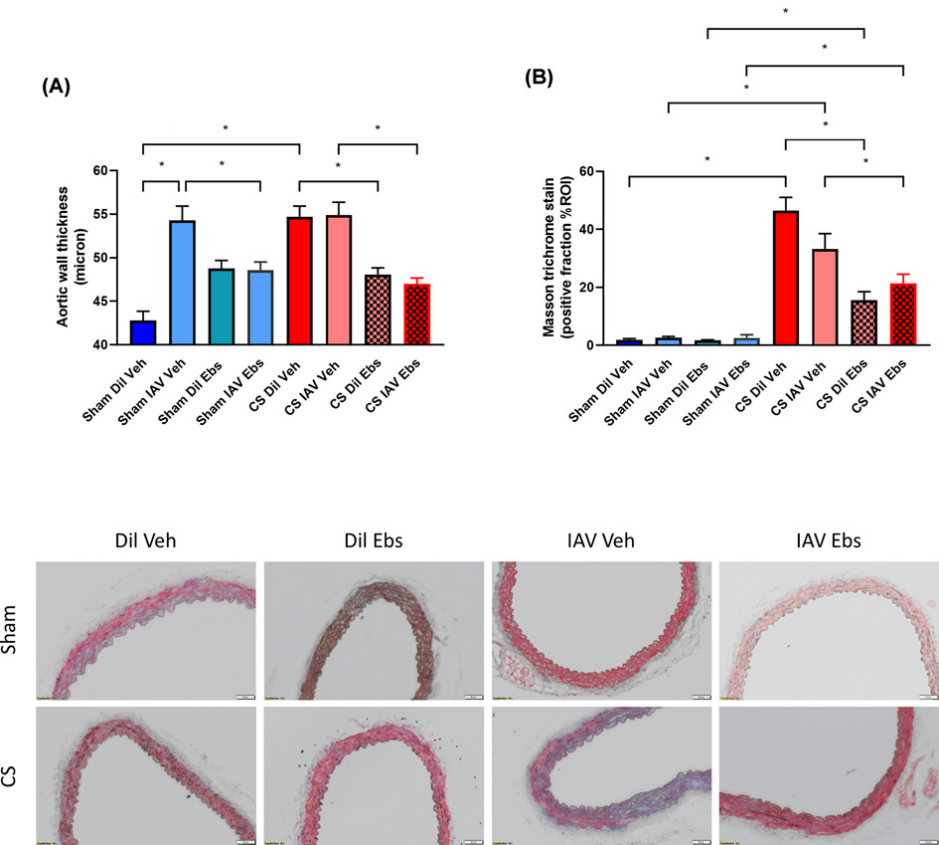
Ebselen treatment prevents aortic remodelling in CS-exposed mice irrespective of IAV infection Aortic wall thickness (**A**) and percentage of positive Masson’s Trichrome stain (**B**) were quantified as an indicator of aortic wall remodelling and collagen deposition, resepctively. Representative photographs of Masson’s Trichrome staining across all experimental groups, with scale bar representing 50 μM. Data expressed as mean ± SEM (*n*=6 mice per group) and analysed by two-way ANOVA with Tukey’s multiple comparisons; * indicates statistical significance *P*<0.05.

We then went on to determine whether there was vascular remodelling in mouse aortae taken from the various experimental groups. There was a significant increase in aortic wall thickness and collagen deposition in mice exposed to CS, however this was not worsened in the presence of IAV infection (Figure 11A,B). Interestingly, ebselen significantly reduced CS-induced aortic wall thickening and collagen deposition (Figure 11A,B).

## Discussion

In the present study, we explored the mechanism driving the worsened cardiovascular outcomes during respiratory pathogen-induced exacerbations of COPD. Using our pre-clinical model of COPD, we investigated the role of AECOPD on vascular function, and the therapeutic potential of ebselen; a National Institute of Health (NIH)/Food and Drug Administration (FDA)-approved antioxidant [32,33], for the concurrent treatment of both the pulmonary and cardiovascular manifestations observed during AECOPD. The results from this study offer proof-of-concept data into the key oxidative mechanisms driving worsened vascular endothelial dysfunction during AECOPD, whilst outlining a novel role of the adaptive immune system.

We have previously shown that mice chronically exposed to CS had impaired vascular function [13]. Consistent with this, the present study showed endothelium-dependent vasodilatory responses to ACh were significantly impaired in mice exposed to CS on both days 3 and 10, when compared with the sham group. CS-exposed mice infected with IAV (mimicking AECOPD) showed an even greater level of endothelial dysfunction at the d3 time point when compared with CS + diluent-treated mice. Interestingly, at the d10 time point CS + IAV-infected mice no longer showed this exacerbated impairment indicating that during this resolution phase of infection, endothelial function is returned to baseline CS levels. The greater endothelial dysfunction observed in the CS + IAV group at d3 compared with the CS + diluent group may be associated with the peak in lung viral load at d2–4 post-infection in mouse and human H1N1 influenza infection, causes enhanced inflammation [21,34–36]. Whilst we did not measure viral load in the lungs of mice in this study, it is not surprising that endothelial dysfunction is returned to baseline CS levels 10-days post-infection as we have previously shown that the IAV has been cleared from the lungs at the 7–10-day timepoint, [21,34]. Moreover, clinical studies have shown that d10 is considered the resolution phase, where a low viral load and reduced symptoms are evident [36–39].

We have previously explored whether IAV that enters the lungs could disseminate into the vasculature to impact endothelial function during pregnancy (Liong et al., 2020 [40]). We found that at d3 post-infection when peak of BALF inflammation is observed, IAV could be detected (via qPCR) in the aorta and this was associated with an enhanced expression of pro-inflammatory and oxidative stress genes within the aorta [40]. We also showed that IAV infection caused profound impairments in vascular function suggesting viral infection *per se* is sufficient to drive extrapulmonary manifestations during pregnancy [40]. In the present study, IAV infection of sham-exposed mice had no adverse effect on endothelium-dependent dilation when compared with the diluent-treated mice at both time points, indicating that IAV infection has no effect on vascular function in healthy mice and this is a CS-dependent phenomena. Consistent with our previous work, there were no alterations to either smooth muscle dilation, suggesting that this was purely a result of a dysfunctional endothelium; a hallmark feature of CVD [10,41,42].

The immune system plays an important role in the pathogenesis of various lung and cardiovascular conditions including atherosclerosis and hypertension, particularly in smokers and COPD patients due to their already heightened inflammatory burden [8,10,42,43]. It is well recognised that both lung inflammation and tissue damage are amplified during AECOPD, and this is largely due to the enhanced recruitment of immune cells to the site of infection where they become hyperactivated, driving the secretion of acute phase and chemoattractant proteins, leading to the degradation of lung tissue via enhanced protease activity and an enhanced oxidative burden [10,44,45]. Prior studies from our laboratory have shown that IAV-infected mice have enhanced pulmonary inflammation, and significantly elevated lung peroxynitrite and superoxide levels compared with uninfected mice [24,46]. We have also shown that superoxide levels are further increased following IAV infection in CS-exposed mice [21]. Therefore, targeting this increased lung inflammation and oxidative stress burden should improve health outcomes in AECOPD. In our study CS exposure caused a significant increase in BALF cellularity which was exacerbated by IAV infection. Interestingly, ebselen treatment altered BALF cell populations, marked by a significant decline in neutrophils and a concomitant increase in macrophage counts.

Pulmonary neutrophilia can be a ‘double-edged sword’, as its recruitment and activation limits viral replication within the lungs, however its prolonged hyperactivation is detrimental to lung integrity due to elastase- and ROS-mediated tissue destruction, thereby limiting respiratory function [47]. Despite the lack of effects on BALF lymphocyte number, ebselen treatment altered neutrophil/macrophage ratio, which effectively switched from a granulocytic neutrophil response to a more macrophage-mediated immunoregulatory response, lowering lung inflammation. As lung inflammation often dictates the functional decline of the lungs in patients experiencing AECOPD [48], a reduced lung inflammation not only should alleviate respiratory dysfunction/symptoms, but also CVD risk. A study by Michaud et al*.* highlighted the detrimental role of CS exposure on human endothelial cells, showing that CS caused a significant reduction in vascular endothelial growth factor activity, endothelial cell migration, enhanced ROS production and caused a decrease in eNOS phosphorylation, negatively regulating NO production [49]. However, antioxidant treatment mitigated the oxidative burden and maintained NO expression *in vitro.* Indeed, our study found that ebselen improved AECOPD-triggered endothelial dysfunction, which is a gateway to more debilitating disorders, such as atherosclerosis and heart failure [50]. Therefore, the respiratory and cardiovascular protective properties of ebselen warrants further clinical investigation.

eNOS catalyses the formation of NO, a key regulator of blood vessel homeostasis and function. The expression of eNOS is significantly reduced in an oxidative manner upon exposure to CS negatively impacting homeostatic balance and vascular integrity [13], with studies by Taddei et al. and Siasos et al. having proven that both oxidative stress in response to CS exposure as well as age-related oxidation reduce NO production/bioavailability [51,52]. The findings from these studies also suggest that eNOS expression is ablated in the presence of ROS, particularly superoxide radicals, therefore setting the rational for eNOS and vascular superoxide quantification. It is well established that NADPH oxidase (NOX) enzymes, play a crucial role in the production of superoxide in both the lungs and circulatory system [46,53,54]. Moreover, NOX enzymes have been implicated in the development of CVD by promoting vascular inflammation, oxidative stress, fibrosis, endothelial dysfunction, and structural remodelling of the arterial wall [55–57]. To make matters worse excessive superoxide can then react with NO, leading to the accumulation of peroxynitrite [10]. This peroxynitrite not only reduces the levels of bioavailable NO leading to the uncoupling of eNOS, it also inactivates various superoxide dismutase isoforms; which are crucial for the disproportionation (the simultaneous oxidation and reduction) of superoxide radicals, driving enhanced superoxide production in a positive feedback-like mechanism [58–60].

Consistent with our previously published work we found that there was a ∼60% suppression in eNOS expression because of CS exposure, when compared with the sham-exposed control mice irrespective of IAV infection [13]. Vascular peroxynitrite expression was then analysed using 3-NT, with the data showing an ∼1.5-fold increase in the vascular oxidative stress burden in all CS-exposed mice when compared with the sham controls, however IAV infection caused no further increase in vascular oxidative stress. This finding suggests that an alternate mechanism was driving the worsened endothelial dysfunction observed CS + IAV mice.

It is well established that T cells play a crucial role in the development of numerous cardiovascular complications, in particular, CD3+ T cell recruitment/adhesion to the aortic wall has been shown to play a pathological role in hypertension, via excessive ROS production and the secretion of pro-inflammatory mediators [61,62]. A study by Laniewski et al. found that during viral infection, there is a significant increase in T cell proliferation which is not completely resolved upon viral clearance, and these T cells remain activated and continue to produce substantial amounts of pro-inflammatory mediators and ROS [63]. This same study also showed that the metalloporphyrin-mimetic (has SOD like activity) significantly decreased levels of T cell expansion in virus-infected mice. Therefore, it is possible that the aortae of CS-exposed mice which have an overexuberant oxidative stress burden, enhanced T cell recruitment into the aortic wall upon IAV infection, driving the worsened vascular function observed during AECOPD. Interestingly, we found that CS exposure or IAV infection alone did not increase T cell number but that CS-exposed IAV-infected mice showed a significant increase in the number of infiltrating CD3+ T cells at both the d3 and d10 time points. The fact that this was only observed in mice exposed to CS confirms the compounding effects viral exacerbations have beyond the respiratory system, recapitulating that of AECOPD patients.

Investigation into vascular remodelling was also conducted within this study, as collagen deposition has been implicated in the development of CVD [10,41,64,65]. Patients with comorbid CVD often develop atherosclerotic lesions and hypertension which is largely due to enhanced collagen deposition within the walls of the vasculature driving vessel hypertrophy [19,66]. Findings from the present study showed there was a significant increase in aortic wall thickness and collagen deposition in mice exposed to CS, however this was not worsened in the presence of IAV infection. Interestingly, ebselen significantly reduced CS-induce aortic wall thickening and collagen deposition.

Having established the CS-dependent nature of IAV infection on vascular dysfunction, we went on to address whether targeting oxidative stress with ebselen may lower lung inflammation and improve vascular function. Treatment with ebselen completely prevented the endothelial dysfunction associated with IAV-induced exacerbations of CS-induced lung inflammation. We believe that this is due to the modulation of both the pulmonary and vascular oxidative stress burden and a subsequent reduction in T-cell expansion/recruitment into the aortic wall. Won et al. showed that GPx-deficient CD4+ T cells produce more intracellular ROS, have a higher proliferation rate, and secrete more pro-inflammatory mediators such as interleukin-2 than wildtype cells [67]. This highlights the importance of GPx as a cellular antioxidant and the therapeutic potential of enhancing cellular antioxidant defence in T cell-mediated immunity. A study by Ahwach et al. explored the effects of ebselen treatment on both human umbilical vein endothelial cells (HUVECs) and human coronary artery endothelial cells (HCAECs) [68], and concluded that hyperglycaemic mice treated with ebselen prevented the induction of diabetes-related oxidative stress, which effectively alleviated endothelial dysfunction and CVD morbidity.

Consistent with the initial findings of the present study, expression of both eNOS and 3-NT was unaltered in response to IAV infection following CS. This then postulates the idea that due to the already enhanced oxidative burden observed in the aortae of mice exposed to CS, IAV infection drives T cell recruitment into the aortic wall in an oxidant-dependent manner, as this phenomenon was not observed in sham-exposed mice. Therefore, these findings highlight the therapeutic potential of antioxidant therapy in the treatment of CVD during AECOPD.

In conclusion, IAV infection further worsens CS-induced endothelial dysfunction in mice. We have also shown for the first time that T cell recruitment into the aortic wall is enhanced in IAV-infected mice following CS exposure suggesting a potential novel mechanism responsible for the worsened vascular outcomes. Moreover, the antioxidant ebselen was able to completely prevent the endothelial dysfunction and enhanced T cell recruitment associated with IAV infection and CS-induced lung inflammation. Therefore, ebselen may be a novel therapeutic for the concurrent treatment of both the pulmonary and cardiovascular manifestations associated with AECOPD.

## Clinical perspectives

- CVD is the leading cause of death in people with COPD, which is further exacerbated during viral infection, enhancing the need for hospitalisation and mortality.
- The present study defines the mechanism driving worsened CVD outcomes in AECOPD and defines a novel therapeutic approach for concurrent treatment of CVD and viral exacerbated COPD.
- Our research shows that oxidative stress and the immune system play a crucial role in manifesting CVD during acute exacerbations. Antioxidant treatment with ebselen may be a feasible way to concurrently treat the pulmonary and cardiovascular manifestations during virus-induced exacerbations of COPD.

## Data Availability

The data that support the findings of the present study are available from the corresponding author upon reasonable request. Some data may not be made available because of privacy or ethical restrictions.

## Abbreviations

3-NT: 3 nitrotyrosine
ACh: acetylcholine
AECOPD: acute exacerbations of chronic obstructive pulmonary disease
ANOVA: analysis of variance
BALF: bronchoalveolar lavage fluid
CD3: cluster of differentiation 3
COPD: chronic obstructive pulmonary disease
CS: cigarette smoke
CVD: cardiovascular disease
Dil: diluent
eNOS/NOS3: endothelial nitric oxide synthase
GPx: glutathione peroxidase
HUVEC: human umbilical vein endothelial cell
IAV: influenza A virus
NO: nitric oxide
NOX: NADPH oxidase
R_MAX_: maximal relaxation
ROS: reactive oxygen species
SEM: standard error of the mean
SNP: sodium nitroprusside
Veh: vehicle

## References

[1] Quaderi S.A. and Hurst J.R. (2018) The unmet global burden of COPD. Glob. Health Epidemiol. Genom. 3, e4 10.1017/gheg.2018.1 29868229 PMC5921960

[2] World Health Organisation (2020) The top 10 causes of death. World Health Organisationhttps//www.who.int/news-room/fact-sheets/detail/the-top-10-causes-of-death.

[3] The Lung Foundation Australia (TLFA) (2017) COPD: the statistics. Health Direct Australiahttp://lungfoundation.com.au/health-professionals/clinical-resources/copd/copd-the-statistics/

[4] Sin D.D. and Man S.F. (2005) Chronic obstructive pulmonary disease as a risk factor for cardiovascular morbidity and mortality. Proc. Am. Thorac. Soc. 2, 8–11 10.1513/pats.200404-032MS 16113462

[5] Lipson D.A., Barnhart F., Brealey N., Brooks J., Criner G.J., Day N.C. et al. (2018) Once-daily single-inhaler triple versus dual therapy in patients with COPD. N. Engl. J. Med. 378, 1671–1680 10.1056/NEJMoa1713901 29668352

[6] Echevarria C., Steer J., Heslop-Marshall K., Stenton S.C., Hickey P.M., Hughes R. et al. (2017) The PEARL score predicts 90-day readmission or death after hospitalisation for acute exacerbation of COPD. Thorax 72, 686–693 10.1136/thoraxjnl-2016-209298 28235886 PMC5537524

[7] Mackay A.J. and Hurst J.R. (2013) COPD exacerbations: causes, prevention, and treatment. Immunol. Allergy Clin. North Am. 33, 95–115 10.1016/j.iac.2012.10.006 23337067

[8] Rabe K.F., Hurst J.R. and Suissa S. (2018) Cardiovascular disease and COPD: dangerous liaisons? Eur. Respir. Rev. 27, 10.1183/16000617.0057-2018 30282634 PMC9488649

[9] Marchetti N., Criner G.J. and Albert R.K. (2013) Preventing acute exacerbations and hospital admissions in COPD. Chest 143, 1444–1454 10.1378/chest.12-1801 23648908

[10] Brassington K., Selemidis S., Bozinovski S. and Vlahos R. (2019) New frontiers in the treatment of comorbid cardiovascular disease in chronic obstructive pulmonary disease. Clin. Sci. (Lond.) 133, 885–904 10.1042/CS20180316 30979844 PMC6465303

[11] Chan S.M.H., Cerni C., Passey S., Seow H.J., Bernardo I., van der Poel C. et al. (2020) Cigarette smoking exacerbates skeletal muscle injury without compromising its regenerative capacity. Am. J. Respir. Cell Mol. Biol. 62, 217–230 10.1165/rcmb.2019-0106OC 31461300

[12] Austin V., Crack P.J., Bozinovski S., Miller A.A. and Vlahos R. (2016) COPD and stroke: are systemic inflammation and oxidative stress the missing links? Clin. Sci. (Lond.) 130, 1039–1050 10.1042/CS20160043 27215677 PMC4876483

[13] Brassington K., Chan S.M.H., Seow H.J., Dobric A., Bozinovski S., Selemidis S. et al. (2021) Ebselen reduces cigarette smoke-induced endothelial dysfunction in mice. Br. J. Pharmacol. 178, 1805–1818 10.1111/bph.15400 33523477 PMC8074626

[14] Bernardo I., Bozinovski S. and Vlahos R. (2015) Targeting oxidant-dependent mechanisms for the treatment of COPD and its comorbidities. Pharmacol. Ther. 155, 60–79 10.1016/j.pharmthera.2015.08.005 26297673

[15] Domej W., Oettl K. and Renner W. (2014) Oxidative stress and free radicals in COPD implications and relevance for treatment. Int. J. Chron. Obstruct. Pulm. Dis. 9, 1207–1224 10.2147/COPD.S51226 PMC4207545 25378921

[16] Duong C., Seow H.J., Bozinovski S., Crack P.J., Anderson G.P. and Vlahos R. (2010) Glutathione peroxidase-1 protects against cigarette smoke-induced lung inflammation in mice. Am. J. Physiol. Lung Cell. Mol. Physiol. 299, L425–L433 10.1152/ajplung.00038.2010 20511341

[17] Takahashi T., Kobayashi S., Fujino N., Suzuki T., Ota C., He M. et al. (2012) Increased circulating endothelial microparticles in COPD patients: a potential biomarker for COPD exacerbation susceptibility. Thorax 67, 1067–1074 10.1136/thoraxjnl-2011-201395 22843558

[18] Bäck M. (2008) Atherosclerosis, COPD and chronic inflammation. Respir. Med. 4, 60–65 10.1016/j.rmedu.2008.01.007

[19] Badimon L., Padro T. and Vilahur G. (2012) Atherosclerosis, platelets and thrombosis in acute ischaemic heart disease. Eur. Heart J. Acute Cardiovasc. Care 1, 60–74 10.1177/2048872612441582 24062891 PMC3760546

[20] Antus B., Paska C., Simon B. and Barta I. (2018) Monitoring antioxidant enzyme activity during exacerbations of chronic obstructive pulmonary disease. COPD 15, 496–502 10.1080/15412555.2018.1535581 30475645

[21] Oostwoud L.C., Gunasinghe P., Seow H.J., Ye J.M., Selemidis S., Bozinovski S. et al. (2016) Apocynin and ebselen reduce influenza A virus-induced lung inflammation in cigarette smoke-exposed mice. Sci. Rep. 6, 20983 10.1038/srep20983 26877172 PMC4753462

[22] Kobayashi N., DeLano F.A. and Schmid-Schonbein G.W. (2005) Oxidative stress promotes endothelial cell apoptosis and loss of microvessels in the spontaneously hypertensive rats. Arterioscler. Thromb. Vasc. Biol. 25, 2114–2121 10.1161/01.ATV.0000178993.13222.f2 16037565

[23] Wong C.H., Bozinovski S., Hertzog P.J., Hickey M.J. and Crack P.J. (2008) Absence of glutathione peroxidase-1 exacerbates cerebral ischemia-reperfusion injury by reducing post-ischemic microvascular perfusion. J. Neurochem. 107, 241–252 10.1111/j.1471-4159.2008.05605.x 18691391

[24] Yatmaz S., Seow H., Gualano R., Wong Z., Stambas J., Selemidis S. et al. (2013) Glutathione peroxidase-1 reduces influenza A virus-induced lung inflammation. Am. J. Respir. Cell Mol. Biol. 48, 17–26 10.1165/rcmb.2011-0345OC 23002098

[25] Belvisi M.G., Haddad E.B., Battram C., Birrell M., Foster M. and Webber S. (2000) Anti-inflammatory properties of ebselen in a model of sephadex-induced lung inflammation. Eur. Respir. J. 15, 579–581 10.1034/j.1399-3003.2000.15.25.x 10759456

[26] Haddad E.B., McCluskie K., Birrell M.A., Dabrowski D., Pecoraro M., Underwood S. et al. (2002) Differential effects of ebselen on neutrophil recruitment, chemokine, and inflammatory mediator expression in a rat model of lipopolysaccharide-induced pulmonary inflammation. J. Immunol. 169, 974–982 10.4049/jimmunol.169.2.974 12097404

[27] Vlahos R. and Bozinovski S. (2014) Recent advances in pre-clinical mouse models of COPD. Clin. Sci. (Lond.) 126, 253–265 10.1042/CS20130182 24144354 PMC3878607

[28] Vlahos R., Bozinovski S., Jones J.E., Powell J., Gras J., Lilja A. et al. (2006) Differential protease, innate immunity, and NF-kappaB induction profiles during lung inflammation induced by subchronic cigarette smoke exposure in mice. Am. J. Physiol. Lung Cell. Mol. Physiol. 290, L931–L945 10.1152/ajplung.00201.2005 16361358

[29] Casanova C., Celli B.R., de-Torres J.P., Martinez-Gonzalez C., Cosio B.G., Pinto-Plata V. et al. (2017) Prevalence of persistent blood eosinophilia: relation to outcomes in patients with COPD. Eur. Respir. J. 50(5), 1701162 10.1183/13993003.01162-2017 29167301

[30] Crisan L., Wong N., Sin D.D. and Lee H.M. (2019) Karma of cardiovascular disease risk factors for prevention and management of major cardiovascular events in the context of acute exacerbations of chronic obstructive pulmonary disease. Front. Cardiovasc. Med. 6, 79 10.3389/fcvm.2019.00079 31294030 PMC6603127

[31] Dransfield M.T., Rowe S.M., Johnson J.E., Bailey W.C. and Gerald L.B. (2008) Use of β blockers and the risk of death in hospitalised patients with acute exacerbations of COPD. Thorax 63, 301–305 10.1136/thx.2007.081893 17951276

[32] Haritha C.V., Sharun K. and Jose B. (2020) Ebselen, a new candidate therapeutic against SARS-CoV-2. Int. J. Surg. 84, 53–56 10.1016/j.ijsu.2020.10.018 33120196 PMC7583587

[33] Garland M., Hryckowian A.J., Tholen M., Bender K.O., Van Treuren W.W., Loscher S. et al. (2020) The clinical drug ebselen attenuates inflammation and promotes microbiome recovery in mice after antibiotic treatment for CDI. Cell Rep. Med. 1(1), 100005 10.1016/j.xcrm.2020.100005 32483557 PMC7263476

[34] Gualano R.C., Hansen M.J., Vlahos R., Jones J.E., Park-Jones R.A., Deliyannis G. et al. (2008) Cigarette smoke worsens lung inflammation and impairs resolution of influenza infection in mice. Respir. Res. 9, 53 10.1186/1465-9921-9-53 18627612 PMC2483272

[35] To K.K., Chan K.H., Li I.W., Tsang T.Y., Tse H., Chan J.F. et al. (2010) Viral load in patients infected with pandemic H1N1 2009 influenza A virus. J. Med. Virol. 82, 1–7 10.1002/jmv.21664 19950247 PMC7167040

[36] Carrat F., Vergu E., Ferguson N.M., Lemaitre M., Cauchemez S., Leach S. et al. (2008) Time lines of infection and disease in human influenza: a review of volunteer challenge studies. Am. J. Epidemiol. 167, 775–785 10.1093/aje/kwm375 18230677

[37] Price I., Mochan-Keef E.D., Swigon D., Ermentrout G.B., Lukens S., Toapanta F.R. et al. (2015) The inflammatory response to influenza A virus (H1N1): an experimental and mathematical study. J. Theor. Biol. 374, 83–93 10.1016/j.jtbi.2015.03.017 25843213 PMC4426089

[38] Hayden F.G., Fritz R., Lobo M.C., Alvord W., Strober W. and Straus S.E. (1998) Local and systemic cytokine responses during experimental human influenza A virus infection. Relation to symptom formation and host defense. J. Clin. Invest. 101, 643–649 10.1172/JCI1355 9449698 PMC508608

[39] Trammell R.A., Liberati T.A. and Toth L.A. (2012) Host genetic background and the innate inflammatory response of lung to influenza virus. Microbes Infect. 14, 50–58 10.1016/j.micinf.2011.08.008 21920449

[40] Liong S., Oseghale O., To E.E., Brassington K., Erlich J.R., Luong R. et al. (2020) Influenza A virus causes maternal and fetal pathology via innate and adaptive vascular inflammation in mice. Proc. Natl. Acad. Sci. U.S.A. 117, 24964–24973 10.1073/pnas.2006905117 32958663 PMC7547222

[41] Kolluru G.K., Bir S.C. and Kevil C.G. (2012) Endothelial dysfunction and diabetes: effects on angiogenesis, vascular remodeling, and wound healing. Int. J. Vasc. Med. 2012, 918267 10.1155/2012/918267 22611498 PMC3348526

[42] Polverino F., Celli B.R. and Owen C.A. (2018) COPD as an endothelial disorder: endothelial injury linking lesions in the lungs and other organs? (2017 Grover Conference Series) Pulm. Circ. 8, 2045894018758528 10.1177/2045894018758528 29468936 PMC5826015

[43] Chandra D., Gupta A., Strollo P.J. Jr, Fuhrman C.R., Leader J.K., Bon J. et al. (2016) Airflow limitation and endothelial dysfunction. Unrelated and independent predictors of atherosclerosis. Am. J. Respir. Crit. Care Med. 194, 38–47 10.1164/rccm.201510-2093OC 26771278 PMC4960631

[44] Barnes P.J. (2016) Inflammatory mechanisms in patients with chronic obstructive pulmonary disease. J. Allergy Clin. Immunol. 138, 16–27 10.1016/j.jaci.2016.05.011 27373322

[45] Hillas G., Perlikos F. and Tzanakis N. (2016) Acute exacerbation of COPD: is it the “stroke of the lungs”? Int. J. Chron. Obstruct. Pulmon. Dis. 11, 1579–1586 10.2147/COPD.S106160 27471380 PMC4948693

[46] Vlahos R., Stambas J., Bozinovski S., Broughton B.R., Drummond G.R. and Selemidis S. (2011) Inhibition of Nox2 oxidase activity ameliorates influenza A virus-induced lung inflammation. PLoS Pathog. 7, e1001271 10.1371/journal.ppat.1001271 21304882 PMC3033375

[47] Vaitkus M., Lavinskiene S., Barkauskiene D., Bieksiene K., Jeroch J. and Sakalauskas R. (2013) Reactive oxygen species in peripheral blood and sputum neutrophils during bacterial and nonbacterial acute exacerbation of chronic obstructive pulmonary disease. Inflammation 36, 1485–1493 10.1007/s10753-013-9690-3 23872721

[48] Stanescu D., Sanna A., Veriter C., Kostianev S., Calcagni P.G., Fabbri L.M. et al. (1996) Airways obstruction, chronic expectoration, and rapid decline of FEV1 in smokers are associated with increased levels of sputum neutrophils. Thorax 51, 267–271 10.1136/thx.51.3.267 8779129 PMC1090637

[49] Michaud S.É., Dussault S., Groleau J., Haddad P. and Rivard A. (2006) Cigarette smoke exposure impairs VEGF-induced endothelial cell migration: role of NO and reactive oxygen species. J. Mol. Cell Cardiol. 41, 275–284 10.1016/j.yjmcc.2006.05.004 16806264

[50] Sun H.J., Wu Z.Y., Nie X.W. and Bian J.S. (2020) Role of endothelial dysfunction in cardiovascular diseases: the link between inflammation and hydrogen sulfide. Front. Pharmacol. 10, 1568 10.3389/fphar.2019.01568 32038245 PMC6985156

[51] Taddei S., Virdis A., Ghiadoni L., Salvetti G., Bernini G., Magagna A. et al. (2001) Age-related reduction of NO availability and oxidative stress in humans. Hypertension 38, 274–279 10.1161/01.HYP.38.2.274 11509489

[52] Siasos G., Tousoulis D., Vlachopoulos C., Antoniades C., Stefanadi E., Ioakeimidis N. et al. (2009) The impact of oral L-arginine supplementation on acute smoking-induced endothelial injury and arterial performance. Am. J. Hypertens. 22, 586–592 10.1038/ajh.2009.57 19300425

[53] Ismaeel A., Brumberg R.S., Kirk J.S., Papoutsi E., Farmer P.J., Bohannon W.T. et al. (2018) Oxidative stress and arterial dysfunction in peripheral artery disease. Antioxidants (Basel) 7(10), 145 10.3390/antiox7100145 30347720 PMC6210426

[54] Vlahos R. and Selemidis S. (2014) NADPH oxidases as novel pharmacologic targets against influenza A virus infection. Mol. Pharmacol. 86, 747–759 10.1124/mol.114.095216 25301784

[55] Sirker A., Zhang M. and Shah A.M. (2011) sNADPH oxidases in cardiovascular disease: insights from in vivo models and clinical studies. Basic Res. Cardiol. 106, 735–747 10.1007/s00395-011-0190-z 21598086 PMC3149671

[56] Zhang W., Bai J., Tian J., Jia L. and Zhou X. (2016) The role of NADPH oxidases in cardiovascular disease. J. Vasc. Med. Surg. 4, 2 10.4172/2329-6925.1000265

[57] Drummond G.R., Selemidis S., Griendling K.K. and Sobey C.G. (2011) Combating oxidative stress in vascular disease: NADPH oxidases as therapeutic targets. Nat. Rev. Drug Discov. 10, 453–471 10.1038/nrd3403 21629295 PMC3361719

[58] Daiber A., Oelze M., Daub S., Steven S., Schuff A., Kroller-Schon S. et al. (2014) Vascular redox signaling, redox switches in endothelial nitric oxide synthase and endothelial dysfunction.In Systems Biology of Free Radicals and Antioxidants, pp. 1177–1211, Springer-Verlag, Berlin Heidelberg 10.1007/978-3-642-30018-9_48

[59] Karbach S., Wenzel P., Waisman A., Munzel T. and Daiber A. (2014) eNOS uncoupling in cardiovascular diseases–the role of oxidative stress and inflammation. Curr. Pharm. Des. 20, 3579–3594 10.2174/13816128113196660748 24180381

[60] Li H. and Forstermann U. (2014) Pharmacological prevention of eNOS uncoupling. Curr. Pharm. Des. 20, 3595–3606 10.2174/13816128113196660749 24180386

[61] Guzik T.J., Hoch N.E., Brown K.A., McCann L.A., Rahman A., Dikalov S. et al. (2007) Role of the T cell in the genesis of angiotensin II induced hypertension and vascular dysfunction. J. Exp. Med. 204, 2449–2460 10.1084/jem.20070657 17875676 PMC2118469

[62] Vogelgesang A., May V.E., Grunwald U., Bakkeboe M., Langner S., Wallaschofski H. et al. (2010) Functional status of peripheral blood T-cells in ischemic stroke patients. PLoS ONE 5, e8718 10.1371/journal.pone.0008718 20090932 PMC2806837

[63] Laniewski N.G. and Grayson J.M. (2004) Antioxidant treatment reduces expansion and contraction of antigen-specific CD8<sup>+</sup> T cells during primary but not secondary viral infection. J. Virol. 78, 11246–11257 15452243 10.1128/JVI.78.20.11246-11257.2004 PMC521823

[64] Alexander M.R., Moehle C.W., Johnson J.L., Yang Z., Lee J.K., Jackson C.L. et al. (2012) Genetic inactivation of IL-1 signaling enhances atherosclerotic plaque instability and reduces outward vessel remodeling in advanced atherosclerosis in mice. J. Clin. Invest. 122, 70–79 10.1172/JCI43713 22201681 PMC3248279

[65] Flamant M., Placier S., Dubroca C., Esposito B., Lopes I., Chatziantoniou C. et al. (2007) Role of matrix metalloproteinases in early hypertensive vascular remodeling. Hypertension 50, 212–218 10.1161/HYPERTENSIONAHA.107.089631 17515450

[66] Di Marco E., Gray S.P., Chew P., Kennedy K., Cooper M.E., Schmidt H.H. et al. (2016) Differential effects of NOX4 and NOX1 on immune cell-mediated inflammation in the aortic sinus of diabetic ApoE-/- mice. Clin. Sci. (Lond.) 130, 1363–1374 10.1042/CS20160249 27190136

[67] Won H.Y., Sohn J.H., Min H.J., Lee K., Woo H.A., Ho Y.S. et al. (2010) Glutathione peroxidase 1 deficiency attenuates allergen-induced airway inflammation by suppressing Th2 and Th17 cell development. Antioxid. Redox Signal. 13, 575–587 10.1089/ars.2009.2989 20367278

[68] Ahwach S.M., Thomas M., Onstead-Haas L., Mooradian A.D. and Haas M.J. (2015) The glutathione mimic ebselen inhibits oxidative stress but not endoplasmic reticulum stress in endothelial cells. Life Sci. 134, 9–15 10.1016/j.lfs.2015.05.004 26006036

